# The Georgia Medical Association

**Published:** 1899-03

**Authors:** Howard J. Williams

**Affiliations:** Macon, Ga.


### THE GEORGIA MEDICAL ASSOCIATION.

MACON, GA., February 18, 1899.

*Editor Atlanta Medical and Surgical Journal.*

DEAR DOCTOR:—I submit to you the following items for an editorial for the March number. Please fix them up neatly, and push the Association's meeting in Macon, April 19th, 20th and 21st.

The meeting will be held in the Academy of Music, a hall large enough to seat all the doctors in the State comfortably, if they wish to come. Every convenience is in the building—committee rooms, telegraph offices, libraries, etc. The stage will be decorated with flowers, and the portraits of Dr. L. D. Ford, first president, and Dr. Crawford W. Long, a first member and the discoverer of ether in surgical practice.

The address of welcome will be delivered by Hon. Emory Speer, judge United States Court. Reply to address of welcome by Dr. DeSaussure Ford, a former president and son of the first president, of Augusta, Ga.

The five living first members, Dr. J. F. Alexander, of Atlanta, Dr. F. M. Brantly, Senoia, Dr. W. L. Jones, Atlanta (L. H. Jones's father), Dr. W. D. Maddox, Monticello, and Dr. C. F. Redding, Zebulon, will be the honored guests of the Association, and occupy seats on the stage, and be otherwise honored.

A large program of valuable papers by members of the association is being prepared, and will be of unusual interest.

A symposium on "Ether Anesthesia" has been arranged (four prominent members of the Association have consented to divide the subject between them, and a good discussion of the subject will follow by others) in honor of the memory of Dr. Crawford W. Long.

Dr. Nicholas Senn, of the Rush Medical College, Chicago, and of recent surgical fame during the late war, will read a valuable paper on "Emergency Surgery."

Dr. Hobart A. Hare, of the Jefferson Medical College, Philadelphia, will read a paper on "Facts Relative to the Medical Complications, Sequela, and Treatment of Typhoid Fever." His reputation as a writer on therapeutics will be a guarantee of the high character of the paper.

Dr. Joseph Price, of Philadelphia, will read a paper on "An Analysis of Appendicitis Operation Methods." As a specialist in abdominal surgery, we can expect a paper of high order.

The discussion of these papers will also be good. And the presence of these men, with first-class papers, I should think would be worth the price of the trip and three days visit to Macon.

A unique souvenir button is being gotten up as a memento of the semi-centennial of the association.

The Committee on Arrangements, the local medical society, and the citizens generally, are arranging to give the association a public reception on the first night (19th), and an entertainment on the second night, while private dinners, breakfasts, and other functions, will be given to make the anniversary one long to be remembered.

The Committee on New Members reports that there will be a very large application for membership. We thought, in 1897, that we had hit the top notch when we took in, at the last Macon meeting, 104 members.

Our seven hotels will give reduced rates ($1.00 to $2.50 per day) to the visiting doctors and their families. The chairman of the Committee on New Members, and others, write that we had better have a list of private boarding houses ready, as the rush will be great.

Reduced rates will be given by the railroads; a lower rate than ever before.

Urge the importance of every member being present, and work for the association, bring the importance of membership to friends who are not members, and let us see if we cannot enroll every regular practitioner in the State at the next meeting. In your columns urge the obligation that every doctor should feel, that he should be a member of his State Medical Society, become interested in its objects and aims, and the corresponding obligation he owes himself of subscribing to the medical journals of the State he lives in.

I should be greatly obliged if you would, through your journal, push the association to the front every month until the meeting comes off.

The preliminary program will not come out in time for your March number, but I will try and arrange for the permanent program to appear in the April issue.

By the way, I forgot to ask you to urge others to write papers, and in this connection I wish you would either print *in extenso*, or refer to Article VIII (relative to papers) in the By-Laws, Transactions 1898, page 252.

Your active cooperation in this matter is earnestly solicited.

Yours truly,

HOWARD J. WILLIAMS.

---

### ASEPSIS IN LARYNGOLOGY AND OTOLOGY.

Dr. McKee (*Larngoscope*, May, 1898) makes the following practical suggestions regarding asepsis in office and clinic work:

1. To sterilize cutting instruments, place in two and a half per cent. carbolic acid solution for fifteen to twenty minutes, and then dip for a few seconds in boiling water.

2. To sterilize trays, dishes for instruments, etc., pour over them a few drops of alcohol and ignite.

3. To sterilize blunt instruments (forceps, spatulæ, etc.), pass through a spirit lamp.

4. To sterilize catheters, antrum cannulæ, etc., boil for a few minutes in a porcelain-lined dish.

5. To sterilize cotton pledgets for wiping out the ear, dip the cotton-wound probe into a saturated alcoholic solution of boracic acid, ignite it, and allow it to burn for a few seconds, extinguishing before the cotton is charred.

6. To preserve needles, keep in pure lysol.

These hints carefully followed will make an excellent working formula for every-day use.

---

***NOTICE.—This JOURNAL and THE INTERNATIONAL JOURNAL OF SURGERY one year for $1.00 cash, to new subscribers. No out-of-town checks accepted unless 10c. is added for cost of cashing.***

